# Editorial Correspondence

**Published:** 1882-12

**Authors:** 


### Editorial Correspondence.

PHILADELPHIA, November 20, 1882.

DEAR JOURNAL—The assertion, that this is the medical and surgical centre of the new world, is looked upon by the members of the profession here as a truism, and a residence of a few weeks in this great city, with a daily round of its colleges and hospitals, is apt to make a stranger a convert to that idea, always excepting the New York city man, who is unwilling to admit that Philadelphia can equally compete with New York in anything pertaining to either learning, arts or commerce. As long, however, as the medical colleges of New York remain simply proprietary concerns, and the number of students, and the amount to be obtained from graduation fees, is in any sense a primary object with their faculties, so long will the unprejudiced medical man, other things being equal, yield the palm to Philadelphia with its one independent university, which has for years bravely insisted upon a full three years' course of study in its halls, and that in the face of the unworthy competition of a college so great and famous in the medical history of the country as "Jefferson." As with Bellevue, so with her, present prosperity and large emoluments to its professors offer too great a temptation, when, on the other hand, nothing is to be gained but the elevation of the profession by the thorough training of candidates for its honors and the weeding out, by a reasonable preliminary examination, of men entirely unqualified for the exercise of its high duties. That the adhesion to the old plan of requiring but two years' attendance upon the lectures is wise, in a "purely business" point of view, is demonstrated by the immense class at "Jefferson"—so large, indeed, that in the lower lecture room, especially, the students occupying the back seats are at a serious disadvantage. It strikes one as ludicrous to see many of these unfortunates using opera glasses, perhaps to see who was lecturing, but they ought also to be provided with ear trumpets, for some of the professors do not speak loud enough to be heard in the distant seats. It is an encouraging sign, however, that, notwithstanding the three years' graded course and the preliminary examination (which is, by the way, not yet sufficiently rigorous), the roomy halls of the "University of Pennsylvania" are well filled with students. Certainly, those availing themselves of its advantages, can hardly fail to acquire a knowledge of medicine which will give them a decided advantage in the race for professional success and honors in the years to come. The teaching strikes an observer as eminently practical and thorough, and the admirable buildings, occupied by the medical department, provide ample accommodations for all departments. The chemical laboratory is exceptionally complete and well appointed, and in addition to this there are large halls, thoroughly furnished with all necessary appliances, for practical work in Histology, Physiology, Pathology, Experimental Therapeutics, Pharmacy, etc.

The dissecting room is peculiarly admirable. The ventilation is perfect, the light abundant, and the tables and floors so constructed that it can be, and is, kept clean and sweet. But the advantages offered by the university are not confined to the undergraduate. It was among the first to establish a post-graduate course. Under the direction of such men as Profs. Pepper, Goodell, H. C. Wood, Duhring, Ashhurst, and others of national reputation, this course has attracted medical men from all sections of the country, and indeed among the many excellent post-graduate schools which have lately been established in New York, Chicago, St. Louis, and elsewhere, it is doubtful if any have the facilities for this kind of instruction that the University of Pennsylvania enjoys. Within a few blocks of the university are located, 1st, the Philadelphia Hospital, with one thousand beds, which is, during the winter months, at least, filled to overflowing, and which provides an unlimited amount of material for clinical instruction; 2d, the University Hospital, an elegant and commodious building, constructed according to the best-established principles of hospital architecture, and provided with all the appliances pertaining to such institutions of the first class. Clinical lectures are delivered daily in its amphitheatre and wards, in addition to the special bed-side instruction to the post-graduate class; 3d, the Children's Hospital, an admirably-conducted institution, which appears to meet with much favor from the good people of this city, the poor little invalids receiving the most careful attention and every possible comfort; 4th, numerous dispensaries, with which the teachers of the post-graduate course are connected, and which are, therefore, open to the members of the class. In addition to these special advantages for clinical instruction, the course is so arranged as to leave the members free to attend the general clinics on Wednesdays and Saturdays, from 9 A. M. to 2 P. M., at the hospitals, which are held by such eminent teachers as Profs. Pepper, Agnew, H. C. Wood, Bartholow, Da Costa, Goodell, Pancoast, and others. Indeed, the only difficulty one experiences here is to find time to avail himself of the opportunities open to him.

An interesting episode at a late clinic at the Philadelphia Hospital was the introduction, by Dr. Pancoast, of a young man who figured in an important surgical operation twenty-four years ago. His face showed a deep scar on the left cheek. "This," said Dr. Pancoast, "was caused by the removal of another child, which, at birth, was attached to his cheek." A photograph was shown, taken when the young man was an infant of seven months, and before the operation. It represented a baby with an imperfectly-developed fœtus (having hands, feet and trunk, but no head) attached to its left cheek, and rather more than half the size of the child. The operation for its removal was performed by the elder Dr. Pancoast at a clinic in Jefferson College, twenty-four years ago. It was witnessed by Drs. Gross, Leidy, Dunglison, and many others, and at that time was considered a very hazardous operation. The section was made with an ecraseur, then a new instrument, the one employed having been brought from Europe by Dr. Pancoast, and the first of the kind ever used in America. The wisdom and success of the operation was demonstrated by the presence of the young man at this clinic, a quarter of a century later.

The monstrosity, upon dissection, was found to be provided with heart and gastro-alimentary tract, as well as the members already referred to.

No little stir was recently excited here by the action of a member of the obstetrical staff of Philadelphia Hospital, in bringing before the class an etherized patient in labor and delivering her, with the aid of forceps. The woman had been in labor nine hours. The os was widely dilated, but the head refused to engage. The application of the forceps was necessary, and no additional risk to the patient was involved by the delivery in the amphitheatre. Nevertheless, the Hospital Committee met and passed resolutions recommending to the Board the summary dismissal of the physician on account of his action. The matter became public and a vast amount of indignation and sentimental gush was indulged in.

The medical men on the Board were this time, however, true to their colors, and the medical staff of the hospital joined in a protest so strong and unanimous that the Board reconsidered its hasty action. It is hard, at least for a medical man, to see why (it being taken for granted that the conditions are such as to involve no additional risk to the woman) there should be any objection to bringing an obstetric case before the class, and none to the every-day occurrence of the various gynecological operations. It has been the custom for years in all the large hospitals and schools in Europe to give this kind of instruction whenever possible, and very properly so, for there is no department of medicine in which it is of so great importance for the welfare of women, and consequently for humanity, that the young graduate should possess a practical knowledge as that of obstetrics. In other departments he is very unlikely to have thrown upon him any very critical and important case, without the possibility of getting professional experience to support and advise him. But the young doctor's first case of obstetrics may be one in which the life of the mother and child will depend upon his knowledge and skill, and if in the country, he may be out of reach of counsel and have to depend wholly upon himself. That objection should be made to clinical teaching in obstetrics *here* is the more extraordinary when we know that in many of our western colleges such clinics are held without objection from any quarter, and are now regarded as an established and necessary feature of obstetric teaching.

The profession here is fortunate in possessing (what we so greatly need in Buffalo) a well-arranged and centrally-located building, known as the "College of Physicians," at which the various medical societies hold their meetings. A very good library occupies one floor and seems to be well supported. I propose to get the details of its management for the possible benefit of our own medical library, now in the formation stage. I was present at a meeting of the "Pathological Society," of which Dr. Tyson is now President. Dr. Nancrede exhibited a mixomatous tumor of the posterior cervical region; Dr. Bruen, a specimen of carcinoma of pancreas and liver, remarkable in that it occurred in a patient 24 years of age; Dr. Tyson, a pair of chronically-inflamed kidneys from a patient who had died of phthisis pulmonalis. Several other specimens were exhibited, not of any particular interest and the "discussion" was not in any way noteworthy. Indeed, the meetings of the Buffalo Association are, as a rule, more spirited and interesting. The younger Gross read his address as retiring President. It was a brief review of the Society's work during his term of office and did not indicate any very great interest in pathological investigation on the part of the professors of the city.

To-day I had the pleasure of witnessing Prof. Goodell make an ovariotomy. The operation was a simple one and there was nothing particularly noteworthy in his method. That he is a skillful and careful operator, goes without saying. He uses the spray solution, 5 per cent., and keeps all the instruments to be employed immersed in a tray of carbolized water. The pedicle in this case was very small and was ligated and returned. A large number of sutures was employed in closing the abdominal wound, the distance between each being less than ½ an inch. No drainage tube was inserted.

The dispensaries, which are numerous, seem to be in possession of abundant means. The great number of people bearing evidences of enjoying a condition very far from poverty, strikes one very forcibly. Indeed, I rarely see one who, unless appearances are deceptive, could not pay for the attendance of a physician. Apparently, however, no questions are asked, and the applicant not only receives advice, but also medicine, free Such a system must seriously injure the younger men here who are not connected with the dispensaries. The only excuse for such a misdirection of medical charity is that the patients presenting themselves are largely utilized for clinical teaching.

I have not spoken of another of the great hospitals of the city, viz., Jefferson College Hospital, because I have not yet had time to pay it a visit. I have attended the lectures of Profs. Bartholow and Da Costa, and I hope to give you my impression in another letter, but already, my dear editors, I imagine I hear the familiar comment, "too long," which usually precedes the consignment of communications to limbo. A. R. D.

